# Influence of Silane Coupling Agent and Anionic Dispersant on the Dispersion Effect of Silicon Carbide Particles

**DOI:** 10.3390/ma17020425

**Published:** 2024-01-15

**Authors:** Zheng Zheng, Min Li, Wenxiao Zhang, Xuhui Zhang, Jiaxiang Liu, Tianyu Yang

**Affiliations:** 1 Beijing Key Laboratory of Electrochemical Process and Technology for Materials, College of Materials Science and Engineering, Beijing University of Chemical Technology, Beijing 100029, China; 18811365676@163.com (Z.Z.); limin9936@163.com (M.L.); xdfzhaichaoyang@163.com (W.Z.); 18811639816@163.com (X.Z.); 2 Shandong Qingzhou Micropowder Co., Ltd., Qingzhou 262500, China; sales@qzwf.cn

**Keywords:** superfine SiC powders, surface modification, silane coupling agent

## Abstract

Silicon carbide (SiC), as a widely used material, has great properties. To improve the flowability of ultrafine silicon carbide slurry, this study used sodium humate, tetramethylammonium hydroxide (TMAH), and N-(β-monoaminoethyl)-γ-aminopropyltrimethyl(ethoxysilane) (KH792) to modify the ultrafine silicon carbide powder produced by Qingzhou Micro Powder Company. The effects of different modifiers on improving the flowability of ultrafine silicon carbide slurry were investigated by means of viscosity tests, sedimentation experiments, and SEM observations. Their modification mechanisms were investigated by means of zeta potential tests, XPS tests, and so on. In this paper, the initial modification of SiC was carried out with KH792, followed by the secondary modification with anionic and cationic modifiers (tetramethylammonium hydroxide and sodium humate), and the optimal modification conditions were investigated by means of a viscosity test, which showed that the lowest viscosity of the modified SiC reached 0.076 Pa·s and that the absolute maximum value of the zeta potential increased from 47.5 at the time of no modification to 63.7 (maximum values) at the time of modification. This means it has an improved surface charge, which improves dispersion. The adsorption results of the modifier on the silicon carbide surface were also demonstrated by the XPS test results.

## 1. Introduction

Silicon carbide (SiC) is an inorganic substance that is mainly synthetic due to its low natural content [1,2]. The most-used method is to put quartz sand, salt, coke and sawdust together into the electric furnace, raise the temperature to 2000 degrees Celsius so that the effective ingredients in the electric furnace (such as silicon dioxide, petroleum coke, etc.) fully react through chemical methods to remove impurities, and a certain degree of screening and crushing of the treatment. Through this method, one can obtain the finished product of silicon carbide powder.

As an advanced material popular in recent years, silicon carbide is utilized in a wide range of aerospace materials, military, semiconductor, electronic circuit components, and other industrial fields because of its prominent thermal and electrical conductivity, high stiffness, remarkable refractoriness, and chemical stability [3,4,5,6,7]. Due to their diversity of uses, silicon carbide ceramics have a variety of molding methods [5,8,9,10,11,12,13], such as injection molding, isostatic molding, roll molding, and slip casting [14,15]. Slip casting has been extensively used in the production of silicon carbide ceramics in modern society due to its advantages of low cost, high productivity, and easy-to-control distinctions [16,17,18,19,20,21,22,23]. Therefore, slip casting is being applied to a wide range of in the production process of silicon carbide ceramics [24].

On the other hand, a sintering process is also required in the preparation of silicon carbide ceramics. In fact, there are various sintering methods for silicon carbide ceramics [25], such as reaction sintering, unpressurized sintering, isostatic pressure sintering, and recrystallization sintering. As an important variety of modern silicon carbide, recrystallized silicon carbide has the advantages of excellent strength, good conductivity, high degree of corrosion resistance, and high temperature resistance and is widely used in semiconductors, aerospace, and other fields. The embryonic density of recrystallized silicon carbide seriously affects its performance, mainly due to its special sintering mechanism—the evaporation condensation mechanism. Therefore, improving the fluidity of the slurry during the injection molding process—that is, decreasing the viscosity (viscosity < 1 pa·s) and increasing the solid content of the slurry (solid content > 50 vol%)—is crucial for improving the properties of recrystallized silicon carbide ceramic products. However, theoretically, the increase in solid phase content is contradictory to the decrease in viscosity because of the small particle size and high surface energy of the ultrafine silicon carbide powder. In the liquid phase, unmodified silicon carbide powder particles are prone to agglomeration between them. Therefore, in the production process of modern ultrafine silicon carbide ceramics, it is important to prepare modified silicon carbide powder that is not easily agglomerated and ultrafine silicon carbide ceramic slurry with good flowability. In fact, for ultrafine recrystallized silicon carbide, it is extremely important to select a modifier for ultrafine silicon carbide powder with the advantages of good performance, environmental friendliness and easy control to improve the performance of ultrafine recrystallized silicon carbide ceramics.

As a widely used modifier in recent years, silane coupling agent is mainly used for the bridging reaction between inorganic surfaces and organic compounds, and its chemical formula is usually RSiX_3_ [26] (X is usually a chlorine group, methoxy group, ethoxy group, methoxyethoxy group, acetoxy group, etc., which can be hydrolyzed to form a silanol group). In recent years, many scholars have studied the viscosity reduction effect of silane coupling agents on ultrafine silicon carbide powders. In addition, tetramethylammonium hydroxide (TMAH), as a strong organic alkaline compound, has been widely used in the fields of polarographic analysis and surface modification of inorganic substances. In the field of ultrafine silicon carbide modification, many scholars have studied the viscosity-reducing effect of TMAH on silicon carbide [27]. In addition, sodium humate is an organic polymer compound that is easily soluble in water and contains functional groups such as benzene rings and carboxyl groups. It has a wide range of applications in the fields of medicine and surface modification [28].

It is because of the excellent viscosity reduction ability of both TMAH and sodium humate that TMAH and sodium humate were used in this paper for compounding with silane coupling agent N-(β-monoaminoethyl)-γ-aminopropyltrimethyl(ethyl)oxy silane (KH792), respectively, and a comparative study was carried out. In fact, this paper provides an innovative report on the study of silane coupling agent compounded with different modifiers such as sodium humate and TMAH to improve the flowability of ultrafine silicon carbide slurry. In order to further investigate the effect of silane coupling agent compound modifier on the surface of ultrafine silicon carbide powder, this paper used N-(β-aminoethyl)-γ-aminopropyltrimethoxysilane (NH_2_CH_2_CH_2_NHCH_2_CH_2_CH_2_Si(OCH_3_)_3_, KH792) compounded with sodium humate (C_9_H_8_Na_2_O_4_) and tetramethylammonium hydroxide (C_4_H_13_NO, TMAH) were compounded to obtain modified ultrafine silicon carbide powder with good fluidity and low viscosity. By comparing the modification effect and mechanism of the above modifiers, a modifier with better modification effect was screened out. On the other hand, the modification mechanism of the above modifiers was also investigated in this paper. We carried out X-ray photoelectron spectroscopy (XRD) analysis and other analytical means on the modified silicon carbide powder to investigate the effect of the modifier on the surface morphology of ultrafine silicon carbide powder and its modification mechanism. On this basis, the modification mechanism and effect of the modifier were summarized and discussed. On the other hand, through the mechanism study, the reasons for the differences in the viscosity reduction effect of different modifiers were analyzed.

## 2. Materials and Method

### 2.1. Materials

Two types of silicon carbide micropowders were used in this paper, one with a larger particle size (D_50_ = 77.22 μm) and the other with a smaller particle size (D_50_ = 1.595 μm). It is also worth mentioning that both silicon carbide powders used in this paper were purchased from Qingzhou Micro Powder Co. (Qingzhou, China).

KH792 and tetramethylammonium hydroxide were purchased from Shanghai McLean Reagent Company (Shanghai, China), and the sodium humate is sourced from a Chinese company, the name of which is Shanghai Yaohua Chemical Corporation (Shanghai, China). In addition, deionized water and 95 vol% ethanol/water solution were used in this paper.

### 2.2. Preparation of Modified SiC Powders

**Figure 1:**
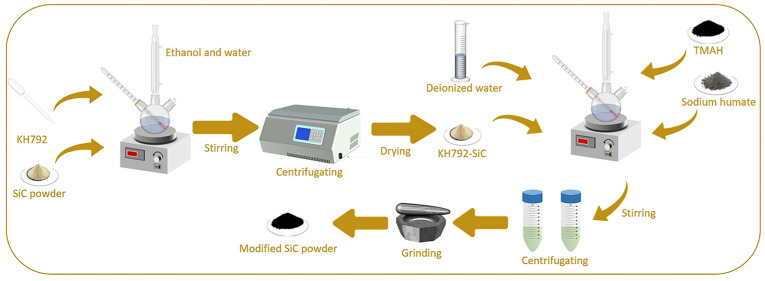
Schematic diagram of silicon carbide powder modification experiment.

As shown in Figure 1, the modification process of ultrafine silicon carbide powder is divided into the following. First, a certain mass of unmodified ultrafine SiC powder, silane coupling agent KH792 and a certain mass of solvent (75% aqueous solution of ethanol) are weighed and mixed into a three-necked flask containing a stirring rotor, and the three-necked flask is placed on a magnetic stirrer for a certain time. The silicon carbide slurry obtained was put into a centrifuge for centrifugation (3600 r, 5 min), and the centrifugal product obtained was washed with deionized water to obtain the washed silicon carbide slurry. The above steps were repeated three times, and the washed slurry was dried to obtain the preliminary modified silicon carbide powder. Then, the preliminary modified silicon carbide powder was mixed with a certain amount of water and modifiers, such as TMAH and sodium humate, and then stirred, washed, and dried—in addition to other steps (specific operation as above)—to obtain modified silicon carbide powder.

### 2.3. Laboratory and Characterization

In this paper, the particle size of the particles was measured using a laser particle size analyzer (LT3600, Linkoptik Instruments Co., Ltd., Zhuhai, China), the viscosity of the SiC slurry was measured using a viscometer (NDJ-85, Shanghai Changji Geological Instrument Co., Shanghai, China), the physical phase compositions of the SiC powders were measured using an X-ray diffractometer (XRD, D/Max-2400, Rigaku, Tokyo, Japan), the morphology of the SiC before and after the modification was observed using a scanning electron microscope (SEM, S-4700, Hitachi, Chiyoda City, Japan), and the functional groups and the chemical compositions on the surface of the SiC particles were measured via X-ray photoelectron spectrometry (XPS, ESCALAB250, ThermoScientific, Waltham, MA, USA). In addition, the stability of SiC powders was investigated via settling experiments, in which certain amounts of modified and unmodified SiC powders were dissolved in a solvent contained in a measuring cylinder (deionized water was used in this paper and the volume concentration of SiC powders was 10 vol%), the initial height of the liquid was recorded (h_0_), and after a period of time of resting, the height of settling was recorded (h) and the calculation of h/h_0_ was used to assess the stability of the slurries.

## 3. Results and Discussions

### 3.1. Characterization of Unmodified Ultrafine SiC Micropowders

**Figure 2:**
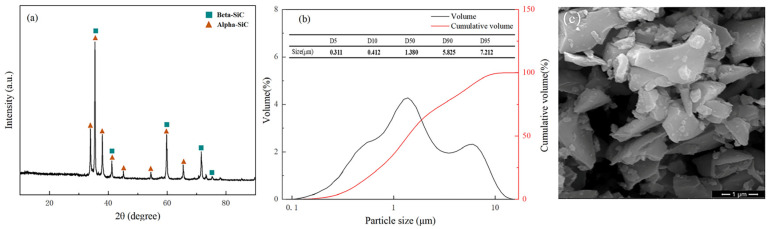
Characterization of unmodified SiC powder, (**a**) XRD pattern, (**b**) particle size analysis (**c**) SEM pattern.

Firstly, this paper performed some characterization analysis of the unmodified SiC powder, as shown in Figure 2. This paper carried out an XRD test, particle size analysis, and an SEM test on the unmodified SiC powder, as can be seen in Figure 2a. The unmodified SiC powder consisted of α-SiC and β-SiC. On the other hand, as can be seen from Figure 2b, the particle size distribution of unmodified SiC powder is uniform and specifically bimodal in distribution, and the D_50_ of unmodified SiC powder used in this paper is 1.38 μm. As can be seen from Figure 2c, there are many agglomerations and larger particle sizes in the microscopic morphology of unmodified SiC powder, which is mainly due to the fact that the unmodified particles have high surface energy when the particle size is small, and there is a tendency for agglomeration between particles through weak interaction forces.

### 3.2. Rheological and Stability Characterization of Modified SiC Slurry

In fact, viscosity test is used in this paper as an important parameter to measure the flowability of ceramic pastes. The lower viscosity of the slurry represents its better flowability. In this paper, several modifiers were selected to modify SiC powder under different conditions and the best modification results were achieved.

**Figure 3:**
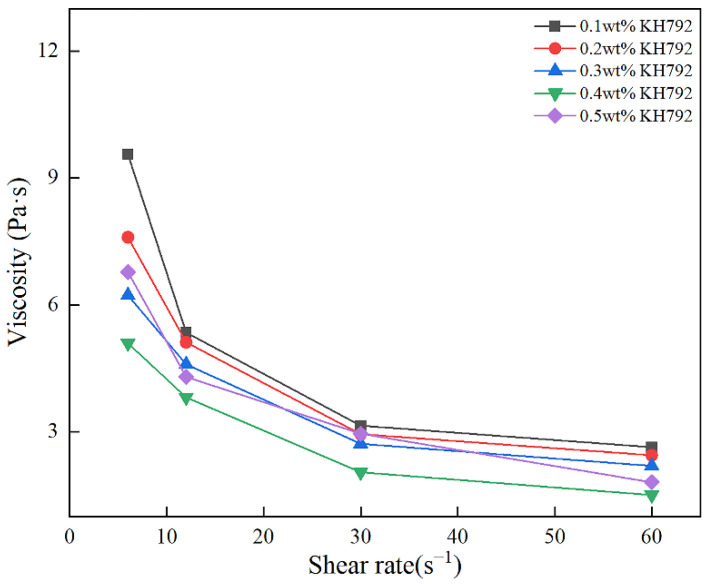
Viscosity of KH792-modified SiC powder slurry at different concentrations.

Figure 3 shows the effect of different KH792 concentrations on the viscosity change of SiC slurry at different rotational speeds when the solid-phase content of SiC is 35 vol%. As can be seen from Figure 3, when the concentration of KH792 is low, the viscosity of SiC slurry is high and its thixotropy is poor, and with the gradual increase in the amount of KH792 added, the viscosity of SiC slurry firstly decreases and then increases. When the addition amount of KH792 is 0.4 wt%, the viscosity of SiC slurry reaches the minimum, and its minimum value is 1.51 Pa·s. The main reason for this phenomenon is actually that when the concentration of KH792 is too low, the modifier fails to adsorb on the surface of the SiC particles in large quantities, resulting in a low encapsulation rate of the modifier. Thus, a good modification effect is not achieved [29]. However, when the concentration of KH792 is too high, the modifier molecules will undergo the reaction of self-entanglement and self-polymerization in the liquid phase, which also leads to its hindering the dispersion of the SiC powder particles, which in turn leads to the high viscosity of the SiC slurry.

**Figure 4:**
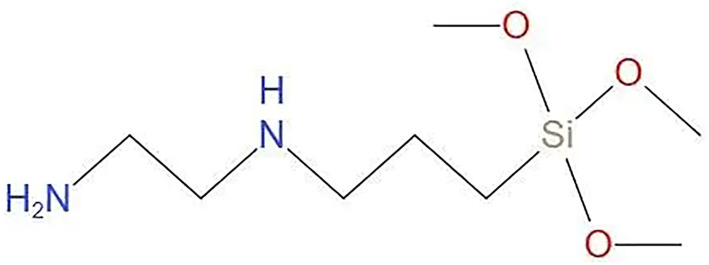
Structural formula of KH792.

**Figure 5:**
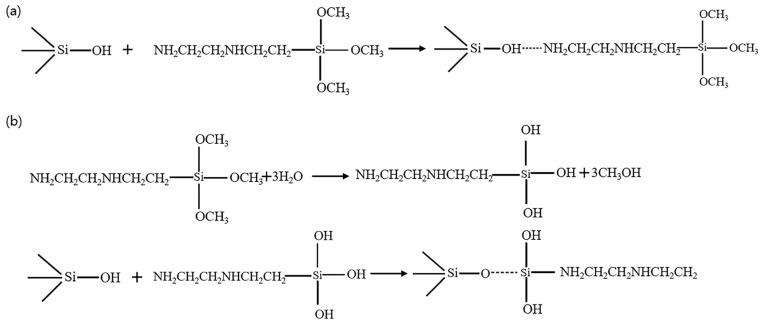
Reaction mechanism equation of KH792 (**a**) Si-O-N binding mechanism and (**b**) Si-O-Si binding mechanism.

It is well known that the surface of SiC generates an oxidized layer after prolonged contact with air, and its main functional groups are Si-OH and Si-O-Si (which depends on the acidity or alkalinity of the environment) [5,30]. Figure 4 shows the molecular structural formula of KH792, the presence of -NH_2_ and Si-OH at its terminus provides the possibility of binding on the surface of SiC particles. When KH792 comes in contact with water, it will undergo a hydrolysis reaction and generate a large amount of Si-OH, which will pass through the surface of the SiC particles in the following two ways. Figure 5 below shows the binding mechanism of the two SiC particles and KH792. As shown in Figure 5a, due to the large number of hydroxyl groups in the oxidized SiC powder particles, the terminal amino group of KH792 undergoes a reversible reaction of dissociation in water, which makes it easier for it to combine with the oxygen atoms in the hydroxyl groups to form a hydrogen bond and then attach to the SiC particles [31]. In addition, as shown in Figure 5b, another interpretation of this paper suggests that KH792 undergoes hydrolysis in water to generate hydroxyl groups and methanol, and in fact, the hydroxyl groups generated by hydrolysis generate hydrogen bonding with Si-OH on the surface of the SiC particles, which in turn allows KH792 to bind on the surface of the SiC particles. Therefore, in this paper, the addition of KH792 at 0.4% was chosen as the result of preliminary modification and subsequent experiments.

**Figure 6:**
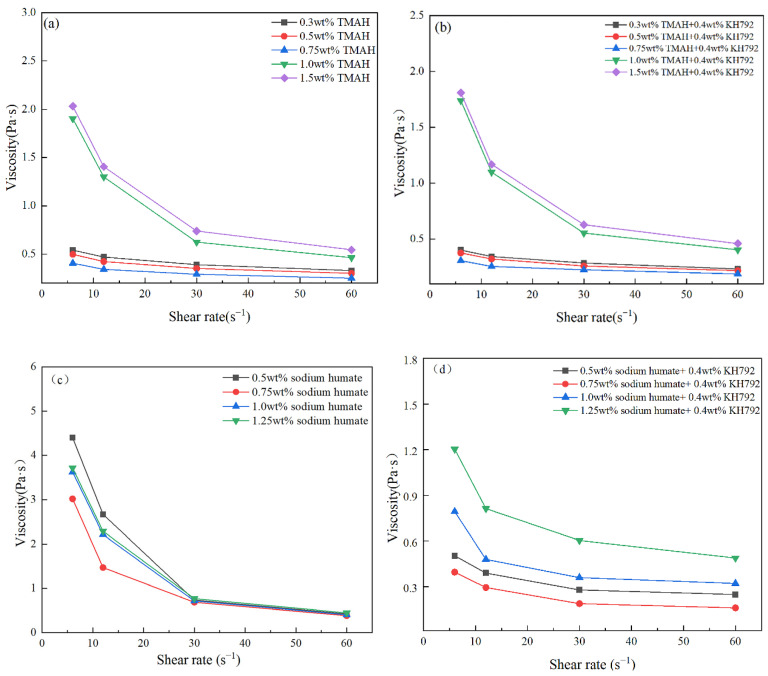
Effect of modifier types and concentration on the viscosity of SiC slurries, (**a**) TMAH, (**b**) TMAH with 0.4 wt% KH792, (**c**) sodium humate, (**d**) sodium humate with 0.4 wt% KH792.

As shown above, 0.4 wt% KH792 was selected as the optimum addition amount, based on which this paper will continue to explore the effect of other modifiers compounded with it on the viscosity of SiC slurry. In order to prepare SiC ceramics with good performance, the solid phase content of the slurry was increased to 50 vol% in this paper while ensuring that the slurry had sufficient fluidity. Following studies on the viscosity of the slurry in this paper will be carried out in the case of a solid-phase content of 50 vol%. The viscosity changes of 50 vol% modified SiC slurry with different types and concentrations of dispersants is shown in Figure 6. As can be seen from Figure 6, with the gradual increase in modifier addition, the viscosities of SiC slurries all showed a tendency of decrease and then increase, with a lowest viscosity of 0.160 Pa·s, which can be obtained via the combined modification of 0.75 wt% sodium humate and 0.4 wt% KH792. However, further increase in the concentration of the modifier leads to an increase in the viscosity of the SiC slurry. Obviously, from Figure 6a,b, the viscosity-reducing effect of TMAH and KH792 complexed with KH792 is better compared to that of TMAH-modified SiC slurry alone, and its minimum viscosity reaches 0.189 Pa·s. From Figure 6c,d, the viscosity-reducing effect of sodium humate complexed with KH792 is also stronger than that of sodium humate alone, and its minimum value of viscosity reached 0.160 Pa·s. The main reason for the above phenomenon is that with KH792 attached to the surface of SiC particles, more active sites appeared on the surface of SiC particles, which made it easier to produce hydrogen bonding with the polar functional groups of the modifier molecules (e.g., -N^+^, etc.), which in turn improved the coverage of the modifier on the surface of SiC and enhanced the mobility of the SiC slurry by means of increasing the spatial site resistance and increasing the surface charge.

**Figure 7:**
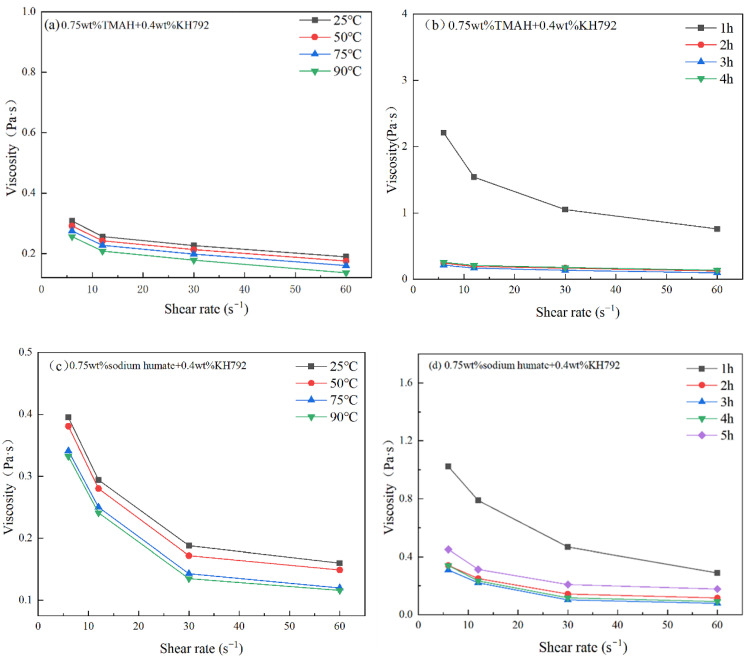
Effect of different modification conditions and types on the viscosity of SiC slurry, (**a**) temperature on TMAH+KH792 co-modified SiC slurry, (**b**) time on TMAH+KH792 modified SiC slurry, (**c**) temperature on sodium humate+KH792 modified SiC slurry, (**d**) time on sodium humate+KH792 modified SiC slurry.

Since all the above modifiers have good viscosity reduction effects for SiC slurry, this paper is further explored for the modification conditions. Figure 7 illustrates the effects of different modifiers on the viscosity of SiC slurry under different conditions of modification temperature and time. It can be seen from Figure 7 that with the increase of modification temperature, the viscosity of SiC slurry modified by different modifiers was reduced, and the modification temperature at which the lowest viscosity was achieved was 90 °C. Furthermore, by fixing the modification temperature at 90 °C, the viscosity of SiC slurry gradually decreased with the increase in modification time, however, when the modification time is too long, the viscosity of SiC slurry increases significantly. In this experiment, the optimal modification condition of TMAH-KH792-SiC slurry was 3 h at 90 °C ambient temperature, the viscosity was 0.096 Pa·s at a rotational speed of 60 rpm, the optimal modification condition of sodium humate-KH792-SiC slurry was 4 h at 90 °C ambient temperature, and the viscosity was 0.076 Pa·s at a rotational speed of 60 rpm. In fact, the reason for this phenomenon is mainly due to the fact that with the gradual increase of the modification temperature, the Brownian motion of the particles in the system is intensified and the speed of the thermal movement of the particles is accelerated. In turn, this leads to a rise in the probability of intermolecular collision, so the modifier molecules can be adsorbed on the surface of the SiC particles more quickly and in greater quantities and thus achieve a better modification effect. In addition. When the modification time is too short, the modifier cannot be effectively adsorbed on the surface of SiC particles, while if the modification time is too long, it will lead to the desorption of part of the modifier from the surface of SiC and self-twisting, self-agglomeration in the system, which will lead to a sharp increase in viscosity.

Zeta potential is an important indicator for evaluating the dispersive properties of slurry systems, and the magnitude of the absolute value of zeta potential reflects the good or bad dispersion performance of powders to a certain extent; the larger the absolute value is, the better the dispersion performance is [28,32,33,34]. In addition, the zeta potential on the surface of the powder particles also realizes the magnitude of the electrostatic repulsion force on the surface of the powder particles, which then helps to analyze the dispersion mechanism [35]. Therefore, this paper chose the SiC powder co-modified with 0.4 wt% KH792 and 0.75 wt% TMAH modified for 4 h at a modification temperature of 90 °C and SiC powder co-modified with 0.4 wt% KH792 and 0.75 wt% sodium humate for comparative analysis.

**Figure 8:**
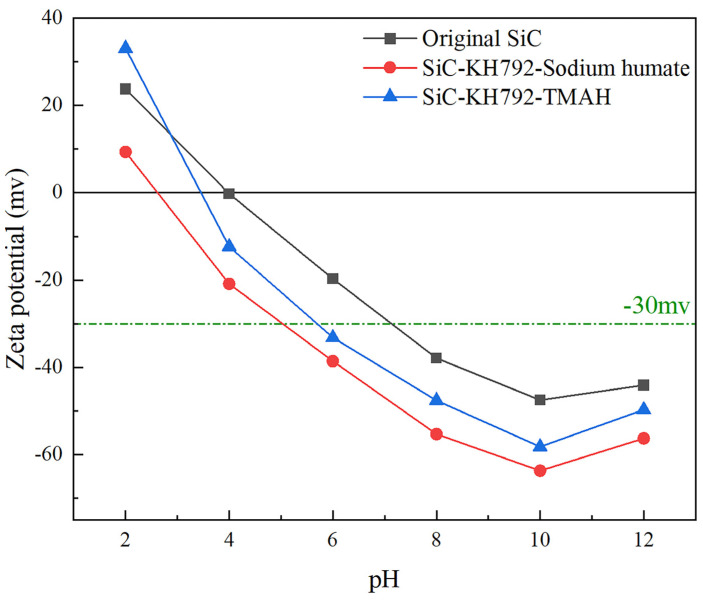
Zeta potential of SiC modified by different modifiers at different pH levels.

As shown in Figure 8, the zeta potential analysis of unmodified SiC slurry, KH792 and TMAH combined, modified SiC slurry and KH792 and sodium humate combined, and modified SiC slurry is shown in Figure 8. It can be seen that the absolute value of the zeta potential of SiC slurry decreases and then increases with the increase in the pH, and the absolute value of the zeta potential decreases again when the pH is too high. The absolute maximum value of zeta potential of TMAH-KH792-SiC slurry was 63.7, and the absolute maximum value of the zeta potential of sodium humate-KH792-SiC slurry was 58.2. The main reason for this phenomenon is that when the pH is too high, the ionic strength in the system is too high, which in turn leads to the adsorbed bilayer on the surface of the SiC particles being compressed [36], making its dispersion worse. On the other hand, it can also be seen from Figure 8 that the zeta potential values of SiC-KH792-TMAH are higher than those of SiC modified with sodium humate. This is due to the fact that when TMAH, as a cationic modifier, is attached to the surface of SiC powder particles, a large amount of N^+^ carried by TMAH is also adsorbed to the surface of SiC, which in turn increases its zeta potential value. In addition, we can see from Figure 8 that at pH > 5, compared to the isoelectric point Ieq = 3.8 of unmodified SiC powder, the modified SiC powder showed higher absolute value of zeta potential, and its isoelectric point Ieq shifted to the left compared to the unmodified SiC powder due to the 90 °C heating that exists in the modification of the SiC powder step in the modification of SiC powder. This resulted in the formation of -SiO- on the surface of SiC under alkaline heating conditions, which in turn resulted in the elevation of the negative charge carried on its surface [37,38]. On the other hand, at pH 4, compared to the unmodified SiC powder, the absolute value of the zeta potential of the silicon carbide slurry co-modified with sodium humate and KH792 was the highest among the three compared to the surface of unmodified TMAH and of that combined with KH792 co-modified SiC particles, and the isoelectric point Ieq shifted more to the left compared to the unmodified SiC powder. The isoelectric point was shifted more to the left, from which it can be inferred that both TMAH and sodium humate improve the electrostatic repulsion between SiC powder particles, which undoubtedly improves the dispersion performance of ultrafine SiC particles in the liquid phase.

**Figure 9:**
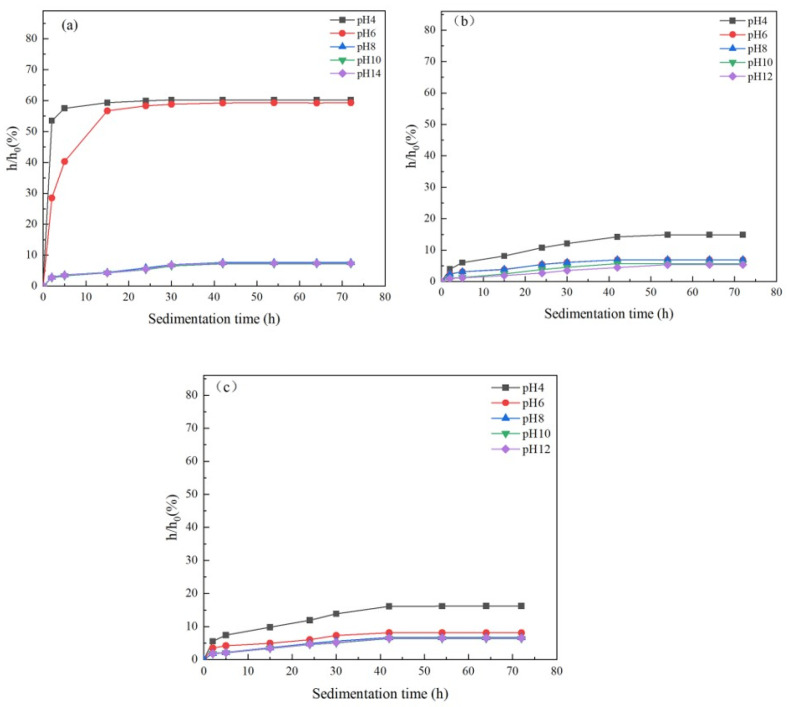
Settling experiments on modified and unmodified SiC, (**a**) settling experiments of unmodified SiC raw powder, (**b**) settling experiments of SiC slurry modified using KH792 in combination with TMAH, (**c**) settling experiments of SiC slurry modified using KH792 in combination with sodium humate.

For the liquid phase stability of SiC slurry before and after modification, a settling experiment was conducted in this paper to characterize it, and in fact, the smaller value of h/h_0_ before and after settling represents the better slurry fluidity. Figure 9a shows the sedimentation experiments of unmodified SiC raw powder. As can be seen from Figure 9a, the sedimentation rate of unmodified SiC raw powder is faster at lower pH, and when pH < 6, h/h_0_ rises extremely fast with the increase in time and then reaches equilibrium at about 25 h, where h/h_0_ is basically unchanged. It then stabilized at about 60, and the change in h/h_0_ was smaller when the pH was above 8. During the period from 0 h to 40 h, h/h_0_ increased gradually with the increase of time, and when the settling time exceeded 40 h, h/h_0_ was basically unchanged. The reason for this phenomenon is mainly related to the zeta potential, as can be seen from Figure 8 above. At pH < 6, the zeta potential of SiC is lower, which means that the charge on the surface of SiC particles is lower and that there is not enough electrostatic repulsion in the liquid phase, which leads to the agglomeration of SiC particles due to the action of van der Waals forces. Additionally, the mass of the agglomerated particles is increased and accelerated under the action of gravity. The mass of the agglomerated particles increases and accelerates under gravity, which ultimately leads to the extreme increase of h/h_0_. As can be seen from Figure 9b,c, compared to the unmodified SiC, the modified SiC raw powder also shows good stability performance at lower pH, and in fact, this is the same as the trend of the absolute value of zeta potential shown in Figure 8. In addition, the SiC slurry in Figure 9c, which has been modified by the combination of sodium humate and KH792, shows a better liquid-phase stability, and at pH = 12, the liquid-phase stability of the SiC slurry is better. Its h/h_0_ was finally stabilized at 5.35% at pH = 12, while the h/h_0_ of the SiC slurry co-modified with TMAH and KH792 was 6.52%, which was attributed to the fact that under alkaline conditions, the sodium humate provided a higher charge density on the surface of SiC, which improved the electrostatic repulsion between SiC particles, counteracted the van der Waals’ forces that led to the agglomeration of the particles, and facilitated the dispersion performance of SiC particles’ dispersion properties, which in turn led to enhanced stability.

**Figure 10:**
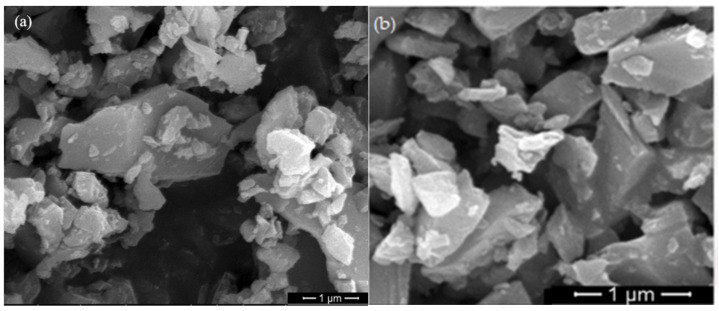
SEM images of modified SiC (**a**) KH792-TMAH-SiC, (**b**) KH792-sodium humate-SiC.

Figure 10 shows the SEM image of modified SiC. Compared to Figure 2c before modification, it can be clearly seen that the agglomeration phenomenon of SiC powder particles is weakened and that the number of SiC with smaller particle size increases in the figure, which also indicates the effect of modification by the modifier.

**Figure 11:**
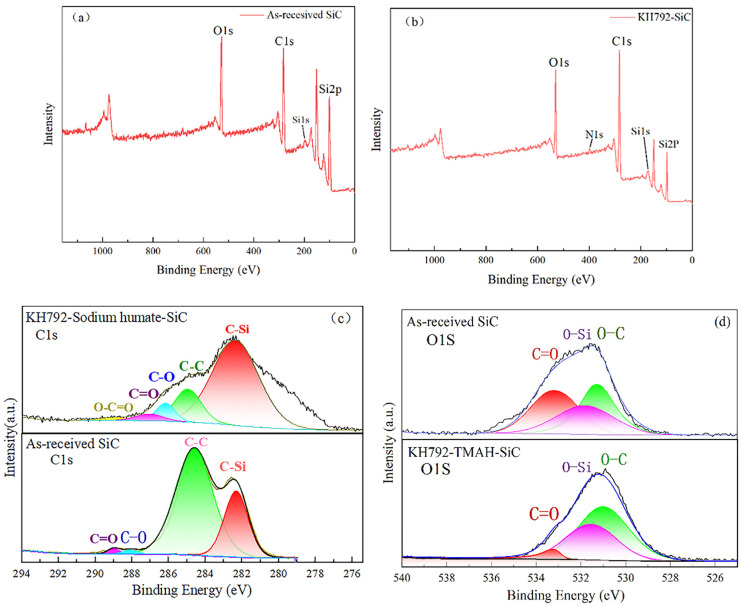
(**a**) XPS wide spectra of unmodified SiC, (**b**) XPS wide spectra of KH792-SiC, (**c**) C1s spectra of modified, and unmodified SiC, (**d**) O1s spectra.

In this paper, XPS analysis was performed for modified and unmodified SiC, and the results are shown in Figure 11. As can be seen from Figure 11a,b, for the unmodified SiC spectra, the peaks of O1s, C1s, Si1s, and Si2p correspond to the peaks at 528.56, 282.18, 176.51, and 101.03 ev, respectively, whereas—as a comparison—the peaks of N1s appeared in the XPS broad spectrum of the SiC modified with KH792 at 399.80 [39,40], which indicates that KH792 was successfully modified to the SiC surface. As can be seen from Figure 11c, for the spectra of C1s of unmodified SiC, the peaks at 282.40, 284.68, 288.18, and 289.08 ev are peaks due to the presence of C-Si, C-C, C-O, and C- [41]. For the jointly modified SiC using KH792 and sodium humate after the joint modification of SiC the jointly modified SiC C1s spectra, a peak of -O-C=O at 298.12 ev was observed, which indicated that sodium humate was successfully bound to the surface of SiC particles [42,43]. On the other hand, Figure 11d shows the O1s spectra of unmodified SiC and SiC modified with TMAH-KH792, which can be seen in Figure 11d, and it can be seen that the SiC modified with KH792-TMAH at 531.25, 531.80, and 533.15 ev showed three peaks corresponding to the peaks generated by C-O, C-Si, and O=C, respectively. As can be seen from Figure 11d, the percentage of C-O area after modification increased from 74% to 86% before modification, which to some extent proved the adsorption of TMAH on the SiC surface.

## 4. Conclusions

In this paper, the silane coupling agent KH792 was compounded with cationic modifier TMAH and anionic modifier sodium humate to modify ultrafine SiC powders, and the modification mechanism was discussed. It was concluded that SiC powders modified with 0.4 wt% KH792 could be prepared with a viscosity of 1.51 Pa·s and a solid phase content of 35 vol%. Under the conditions of TMAH addition of 0.75 wt%, modification temperature of 90 °C, and modification time of 3 h, the modification effect of TMAH-KH792-SiC was the best, and the viscosity of 50 vol% SiC slurry was 0.096 Pa·s. Under the conditions of sodium humate addition of 0.75 wt%, temperature of 90 °C, and modification time of 4 h, the sodium humate KH792-SiC had a minimum viscosity of 0.076 Pa·s. The above modification mainly relies on increasing the electrostatic resistivity effect to promote the dispersion of SiC particles.

## Data Availability

The data presented in this study are available on request from the corresponding author (accurately indicate status).

## References

[1] Chen S., Shang M., Wang L., Yang Z., Gao F., Zheng J., Yang W. (2017). Superior B-Doped SiC Nanowire Flexible Field Emitters: Ultra-Low Turn-On Fields and Robust Stabilities against Harsh Environments. ACS Appl. Mater. Interfaces.

[2] Eom J.-H., Kim Y.-W., Raju S. (2013). Processing and properties of macroporous silicon carbide ceramics: A review. J. Asian Ceram. Soc..

[3] Kota N., Charan M.S., Laha T., Roy S. (2022). Review on development of metal/ceramic interpenetrating phase composites and critical analysis of their properties. Ceram. Int..

[4] Gryn S., Nychyporuk T., Bezverkhyy I., Korytko D., Iablokov V., Lysenko V., Alekseev S. (2018). Mesoporous SiC with Potential Catalytic Application by Electrochemical Dissolution of Polycrystalline 3C-SiC. ACS Appl. Nano Mater..

[5] Kumar P.V., Gupta G.S. (2002). Study of formation of silicon carbide in the Acheson process. Steel Res..

[6] Monteverde F., Scatteia L. (2007). Resistance to Thermal Shock and to Oxidation of Metal Diborides–SiC Ceramics for Aerospace Application. J. Am. Ceram. Soc..

[7] Duong-Viet C., Ba H., El-Berrichi Z., Nhut J.M., Ledoux M.J., Liu Y.F., Pham-Huu C. (2016). Silicon carbide foam as a porous support platform for catalytic applications. New J. Chem..

[8] Akatsu T., Nakanishi S., Tanabe Y. (2013). Toughening enhanced at elevated temperatures in an alumina/zirconia dual-phase matrix composite reinforced with silicon carbide whiskers. J. Eur. Ceram. Soc..

[9] Bai C.Y., Deng X.Y., Li J.B. (2014). Fabrication and properties of cordierite–mullite bonded porous SiC ceramics. Ceram. Int..

[10] Chae S.H., Kim Y.W., Song I.H. (2009). Porosity control of porous silicon carbide ceramics. J. Eur. Ceram. Soc..

[11] Chen W.W., Miyamoto Y. (2014). Fabrication of porous silicon carbide ceramics with high porosity and highstrength. J. Eur. Ceram. Soc..

[12] Ding S., Zhu S., Zeng Y. (2006). Effect of Y_2_O_3_ addition on the properties of reaction-bonded porous SiC ceramics. Ceram. Int..

[13] Guicciardi S., Silverstroni L., Nygren M., Sciti D. (2010). Microstructure and Toughening Mechanisms in Spark Plasma-Sintered ZrB2Ceramics Reinforced by SiC Whiskers or SiC-Chopped Fibers. J. Am. Ceram. Soc..

[14] Barati A., Kokabi M., Famili M.H.N. (2003). Drying of gelcast ceramic parts via the liquid desiccant method. J. Eur. Ceram. Soc..

[15] Zhang C., Huang X., Yin Y., Xia F., Dai J., Zhu Z. (2009). Preparation of boron carbide–aluminum composites by non-aqueous gelcasting. Ceram. Int..

[16] Liu J., Song Y., Wu W., Wang X., Zhou Q., Niu G., Liu P. (2023). Giant permittivity in (Nb_0_._5_La_0_._5_)_x_Ti_1−x_O_2_ ceramics prepared by slip casting in a strong magnetic field. J. Am. Ceram. Soc..

[17] Ayode Otitoju T., Ugochukwu Okoye P., Chen G., Li Y., Onyeka Okoye M., Li S. (2020). Advanced ceramic components: Materials, fabrication, and applications. J. Ind. Eng. Chem..

[18] Cesconeto F.R., Frade J.R. (2020). Cellular ceramics by slip casting of emulsified suspensions. J. Eur. Ceram. Soc..

[19] Evans J.R.G. (2008). Seventy ways to make ceramics. J. Eur. Ceram. Soc..

[20] Nicolás G.O., Yasnó J.P., Gamba M., Lorenzo G., Suárez G. (2023). Dense m-Li_2_ZrO_3_ formed by aqueous slip casting technique: Colloidal and rheological characterization. Ceram. Int..

[21] Marcin W., Justyna Z., Robert K., Paulina P., Radosław Ż., Anna W., Lucjan Ś. (2022). Study on Manufacturing via Slip Casting and Properties of Alumina-Titanium Composite Enhanced by Thialite Phase. Materials.

[22] Kashkarov E., Krinitcyn M., Dyussambayev A., Pirozhkov A., Koptsev M. (2022). Structure and Properties of Porous Ti3AlC2-Doped Al_2_O_3_ Composites Obtained by Slip Casting Method for Membrane Application. Materials.

[23] Raju P., Khanra A.K., Suresh M.B., Rao Y.S., Johnson R. (2022). Pressure slip cast processing of alumina (Al_2_O_3_) products and comparative evaluation of mechanical properties. Adv. Appl. Ceram..

[24] Omatete O.O., Janney M.A., Nunn S.D. (1997). Gelcasting: From laboratory development toward industrial production. J. Eur. Ceram. Soc..

[25] Amirkhanyan N., Kirakosyan H., Zakaryan M., Zurnachyan A., Rodriguez M.A., Abovyan L., Aydinyan S. (2023). Sintering of silicon carbide obtained by combustion synthesis. Ceram. Int..

[26] Björkegren S.M.S., Nordstierna L., Törncrona A., Persson M.E., Palmqvist A.E.C. (2015). Surface activity and flocculation behavior of polyethylene glycol-functionalized silica nanoparticles. J. Colloid. Interface Sci..

[27] Yang J., Yu J., Huang Y. (2011). Recent developments in gelcasting of ceramics. J. Eur. Ceram. Soc..

[28] Liu Y., Liu J., Yang T. (2018). Surface Modification of SiC Powder with Sodium Humate: Adsorption Kinetics, Equilibrium, and Mechanism. Langmuir.

[29] Tian C., Huang X., Guo W., Gao P., Xiao H. (2020). Preparation of SiC porous ceramics by a novel gelcasting method assisted with surface modification. Ceram. Int..

[30] Presser V., Nickel K.G. (2008). Silica on Silicon Carbide. Crit. Rev. Solid. State Mater. Sci..

[31] Romero C.P., Jeldres R.I., Quezada G.R., Concha F., Toledo P.G. (2018). Zeta potential and viscosity of colloidal silica suspensions: Effect of seawater salts, pH, flocculant, and shear rate. Colloids Surf. A Physicochem. Eng. Asp..

[32] Zhang J., Jiang D., Lin Q. (2005). Poly(Vinyl Pyrrolidone), a Dispersant for Non-Aqueous Processing of Silicon Carbide. J. Am. Ceram. Soc..

[33] Shang X., Zhu Y., Li Z. (2016). Dispersion of silicon carbide in poly alpha olefins-6 and trimethylopropane trioleate. Colloids Surf. A Physicochem. Eng. Asp..

[34] Liang H., Pang X., Xu M., Xu T. (2007). Dispersion mechanisms of aqueous silicon nitride suspensions at high solid loading. Mater. Sci. Eng. A.

[35] Zhang Y., Binner J. (2002). In Situ Surface Modification of Silicon Carbide Particles Using Al^3+^ Complexes and Polyelectrolytes in Aqueous Suspensions. J. Am. Ceram. Soc..

[36] Li W., Chen P., Gu M., Jin Y. (2004). Effect of TMAH on rheological behavior of SiC aqueous suspension. J. Eur. Ceram. Soc..

[37] Wołowicz A., Staszak K. (2020). Study of surface properties of aqueous solutions of sodium dodecyl sulfate in the presence of hydrochloric acid and heavy metal ions. J. Mol. Liq..

[38] Gnyla J., Gubernat A., Zych Ł., Nocuń M., Góral Z., Lach R. (2020). Influence of TMAH and NaOH on the stability of SiC aqueous suspensions. Ceram. Int..

[39] Li P., Wang Z., Liu Y., Zhao S., Wang J., Wang S. (2015). A synergistic strategy via the combination of multiple functional groups into membranes towards superior CO_2_ separation performances. J. Membr. Sci..

[40] Qiu L., Zou K., Xu G. (2013). Investigation on the sulfur state and phase transformation of spent and regenerated S zorb sorbents using XPS and XRD. Appl. Surf. Sci..

[41] Shang X., Zhu Y., Li Z. (2017). Surface modification of silicon carbide with silane coupling agent and hexadecyl iodiele. Appl. Surf. Sci..

[42] Xia M.-M., Dong G.-M., Yang R.-J., Li X.-C., Chen Q. (2020). Study on fluorescence interaction between humic acid and PAHs based on two-dimensional correlation spectroscopy. J. Mol. Struct..

[43] Dou G., Jiang Z. (2019). Sodium humate as an effective inhibitor of low-temperature coal oxidation. Thermochim. Acta.

